# Telomere Length in a South African Population Co-Infected with HIV and Helminths

**DOI:** 10.3390/cimb46070409

**Published:** 2024-07-02

**Authors:** Engelinah D. Macamo, Zilungile L. Mkhize-Kwitshana, Zamathombeni Duma, Julian Mthombeni, Pragalathan Naidoo

**Affiliations:** 1 Department of Medical Microbiology, School of Laboratory Medicine and Medical Sciences, College of Health Sciences, Nelson R. Mandela Medical School Campus, University of KwaZulu-Natal, Durban 4001, South Africa; macamoengelinah99@gmail.com (E.D.M.); zamathombeniduma@gmail.com (Z.D.);; 2 Division of Research Capacity Development (RCD), South African Medical Research Council (SAMRC), Tygerberg, Cape Town 7505, South Africa; 3 Department of Biomedical Sciences, Doorfontein Campus, University of Johannesburg, Johannesburg 2028, South Africa; 4 Biomedical Sciences Department of Life and Consumer Sciences, College of Agriculture and Environmental Sciences, University of South Africa, Florida Campus, Johannesburg 1710, South Africa

**Keywords:** HIV, helminths, co-infection, telomere length shortening, biological ageing

## Abstract

Biological ageing refers to the gradual decrease in physiological functions, resulting in immune senescence, cellular damage and apoptosis. Telomere length is a biomarker of biological ageing. Limited studies have associated shorter telomere length with HIV and parasite single infections, with no studies reporting the association of HIV and parasite co-infection with telomere length. The study aimed to investigate whether telomere length shortening is accelerated in a South African population co-infected with HIV and helminths compared to participants singly infected with either HIV or helminths. Additionally, telomere length data were compared with participants’ biochemical and full blood count parameters. A total of 200 participants were in groups of uninfected control, HIV single infection, helminth single infection and HIV and helminth co-infection groups. Relative telomere length (RTL) was determined using Real-Time PCR and associated with biochemical and full blood count parameters using multivariate regression analysis models that were adjusted for confounders. The uninfected control group was used as a reference group. The uninfected control group had the highest mean RTL (1.21 ± 0.53) while the HIV-infected (0.96 ± 0.42) and co-infected (0.93 ± 0.41) groups had similar RTLs, and lastly, the helminth-infected group (0.83 ± 0.33) had the lowest RTL (*p* = 0.0002). When compared to the uninfected control group, a significant association between RTL and biochemical parameters, including blood iron (β = −0.48), ferritin (β = −0.48), transferrin saturation (β = −0.57), transferrin (β = −0.57), phosphate (β = −0.47), vitamin A (β = −0.49) and C-reactive protein (β = −0.52) were noted in the co-infected group (*p* < 0.05). In addition, a significant association between RTL and full blood count, including (β = −0.47), haematocrit (β = −0.46), mean corpuscular volume (β = −0.47), lymphocytes (β = −0.45), mean corpuscular haemoglobin concentration (β = −0.45), red cell distribution width (β = −0.47), monocytes (β = −0.45), eosinophils (β = −0.45), basophils (β = −0.44) and transferrin saturation (β = −0.57) were also noted in the co-infected group (*p* < 0.05). Accelerated biological ageing, as indicated by telomere length shortening, is associated with HIV and helminth co-infections.

## 1. Introduction

Helminthiasis is one of the neglected tropical diseases, infecting close to 2 billion individuals worldwide and is highly prevalent in Sub-Saharan Africa. Parasitic intestinal worms are also part of the helminths. The infections are highly prevalent in the tropics and subtropics of developing countries among communities exposed to poor sanitation and hygiene and with a lack of clean water supply and limited access to health services [1]. In humans, helminth infections are mainly caused by *Ascaris lumbricoides* (*A. lumbricoides*), *Schistosoma* spp., *Trichuris trichiura*, *Necator americanus*; *Ancylostoma duodenale*, *Strongyloides* spp. [2].

According to the World Health Organization (WHO), there are over 39 million HIV-infected individuals worldwide, with Sub-Saharan Africa having over 25.6 million cases. Notably, South Africa (SA) has the world’s highest HIV prevalence among Sub-Saharan African countries, with an estimated 7.5 million HIV infection cases reported in 2022. There is an overlapping geographical distribution of HIV and helminth infections, causing frequent co-infections in the Sub-Saharan Africa region [3,4].

As HIV progresses, the host’s immune system becomes more activated, and the immunological profile changes from being primarily T helper type 1 (Th1) to primarily T helper type 2 (Th2) response. Likewise, helminth infection induces a predominance of Th2 and chronic immune activation [5,6]. The Th1 immune response, which induces an inflammatory reaction, plays a crucial role in the elimination of intracellular infections [7]. For example, during HIV infection, IL-12 promotes the differentiation of naïve T cells to Th1 CD4+, which triggers a Th1 response that secretes pro-inflammatory cytokines such as IL-2, interferon (INF)-γ, tumour necrosis factor (TNF)-α, and macrophage inflammatory protein (MIP)-1α/1β [8]. On the other hand, extracellular infections induce a type 2 helper T-cell (Th2) response. This results in the secretion of cytokines that promote antibody synthesis. The large extracellular helminth infection induces a strong, modified Th2 characterised by the production of cytokines such as interleukin-4 (IL-4), IL-5, and IL-13 [9]. It has been proposed that intestinal helminth infections increase susceptibility to HIV infection and HIV replication in helminth-infected individuals due to persistent immune activation, upregulation of Th2 and regulatory Th3 responses [4,10,11]. Chronic multiple infections like HIV and helminths and continuous antigen stimulation cause T-cell immune exhaustion [12].

The gradual and persistent loss in the functioning of an organism’s cells, tissues, and organs over time is referred to as biological ageing [13]. This phenomenon can be influenced by a variety of factors, including genetics, unhealthy lifestyles [14], socio-demographic environmental factors [15], infection and disease status [16], malnutrition and anaemia [17], and medication and drug usage [18], all of which have been associated with telomere shortening. Telomere length is one of the biomarkers of biological ageing [19]. Although it is not clear how T-cell loss might contribute to biological ageing, the immune system is recognised to play an important role in regulating tissue homeostasis and preventing chronic inflammation, which is also associated with biological ageing [20,21]. Chronic inflammation can lead to the accumulation of damaged cells in tissues, which can accelerate biological ageing. As a result, immune T-cell depletion as a result of chronic inflammation and tissue damage may indirectly lead to biological ageing [22].

Telomeres are protective caps on chromosomes that are made up of DNA sequences. As the cells replicate, the telomeres shorten, resulting in programmed cell death or cell-cycle arrest [23]. Although infections like HIV [16,24,25] and parasites [26,27,28] have been proven to cause telomere shortening and biological ageing in humans, there are no studies reporting whether telomere length shortening is further accelerated during HIV and helminth co-infection.

Studies have reported South African (SA) individuals infected with HIV and helminths to have lower vitamin A, calcium and albumin [29]. To date, there are no studies that explored the association between telomere length and full haematological and or biochemical parameters in HIV and helminth singly or dually infected individuals. Hence, the present study aimed to determine whether telomere length shortening is accelerated in a South African population co-infected with HIV and helminths compared to those uninfected or singly infected with either HIV or helminths. The association between telomere length and haematological plus biochemical parameters was also determined.

## 2. Methodology

### 2.1. Design of the Study and Recruitment of Participants

The current study was part of a main project that aimed to investigate the effects of helminth and HIV co-infections, which enrolled adults (18 years and above; n = 414). Essentially, participants in the study were clinic attendees in primary healthcare facilities that also offer HIV testing and counselling. Others were either clinic personnel or community people who had been notified about the study or were accompanying patients to the clinic. A detailed account of the study setting and participant recruitment has been published previously [30].

For the main study, the estimated prevalence of helminths as determined by stool microscopy in adults in KwaZulu-Natal (KZN) province of SÁ is approximately 36% [31]. To find a sample size that is able to detect differences in proportions between groups with 80% power at *p* < 0.05 significance, the sample size was calculated as 354. To allow for attrition, a total of 421 participants were recruited. For this study, a purposive sampling of 200 participants was performed, owing to laboratory analysis cost implications. The 200 participants were divided into 50 per group on the basis of HIV and helminth infections. This was based on the suggestion that for medium to large effect size for an ordinary study, 30 participants per group can lead to about 80% power [32]. Therefore, the group size of 50 participants in the current study is adequate.

### 2.2. Blood Collection, Haematological and Biochemical Analyses and HIV Detection

Vacuette^®^ EDTA and serum separator blood tubes were used to collect whole blood from all participants and were taken to Neuberg Global Laboratory, a South African National Accreditation System (SANAS) accredited diagnostic laboratory for (i) haematological/full blood count analysis (red cell count, haemoglobin, haematocrit, mean corpuscular volume, mean corpuscular haemoglobin, mean corpuscular haemoglobin concentration, platelets, red cell distribution width, white cell count, neutrophils, lymphocytes, monocytes, eosinophils and basophils) using the Sysmex XN-1000™ Haematology Analyzer (Sysmex, Europe, Norderstedt, Germany); (ii) biochemical analysis (blood iron, ferritin, transferrin, transferrin saturation, calcium, total protein, phosphate, magnesium, vitamin A, albumin, zinc, C-reactive protein, and total IgE) using the UniCel DxC 600i Chemistry Analyser (Beckman Coulter, Brea, CA, USA); (iii) CD4 count analysis; and (iv) HIV viral load analysis. Participant HIV status was confirmed using the Alere Determine^TM^ HIV-1/2 Ag/Ab Combo fast test (Orgenics Ltd., Yavne, Israel). The ICT HIV-1/2 Ag/Ab test kit (ICT Diagnostics, Cape Town, South Africa) was used to confirm inconclusive results.

### 2.3. Stool Collection and Detection of Parasites

Stool samples were collected from all participants and processed for coproscopy using the Kato–Katz (Sterlitech: Auburn, Washington, DC, USA) quantification technique and the Mini Parasep^®^SF modified faecal parasite concentrator technique (Apacor Ltd., Wokingham, UK). Serum samples were sent to the Allergology and Clinical Immunology unit diagnostic research laboratory, Groote Schuur Hospital, University of Cape Town, for the serological detection of *A. lumbricoides*-specific IgE (>0.35 kU/L) and IgG4 (>0.15 kU/L) levels using the Phadia™ 200 instrument (Thermo Fisher Scientific: Phadia AB, Uppsala, Sweden). A detailed account can be found in a previously published article [30].

The 200 participants were purposively selected from the main cohort according to HIV and helminth infection status and grouped as follows: (i) uninfected controls (no HIV and helminth infections) (n = 50), (ii) HIV-infected only group (n = 50), (iii) helminth-infected only group (n = 50), and (iv) HIV and helminth co-infected groups (n = 50).

### 2.4. Leukocyte Telomere Length Assay

The Quick-DNA^TM^ Miniprep Plus isolation kit (Zymo Research, Irvine, CA, USA, Catalogue No: D4069) was used to isolate DNA from blood serum samples. The concentration of the isolated DNA was measured using the Nanodrop2000 spectrophotometer (Thermo Fisher Scientific). DNA samples with an Abs260 nm/Abs280 nm ratio (indicator of protein and lipid contamination) of 1.8–2.0 and an Abs260 nm/Abs230 nm ratio (indicator of solvent contamination) of 1.8–2.2 were considered pure. Thereafter, DNA samples were standardised to 10 ng/µL and stored at −20 °C until use for the telomere length assay.

The leukocyte telomere assay was performed as described by [33]. The Applied Biosystems 7500 Real-Time PCR machine and Applied Biosystems Quant Studio^TM^ Real-Time PCR Software v2.3 (Thermo Fisher Scientific) were used to perform the assay and analyse the data, respectively.

The 10 µL Real-Time PCR reaction mixture in each of the Applied Biosystems MicroAmp^TM^ Optical 96-well PCR reaction plates (Thermo Fisher Scientific) consisted of 4 µL of Applied Biosystems PowerUp^TM^ SYBR Green (Thermo Fisher Scientific), 2 µL of each forward and reverse primers (human 36B4 single copy gene-specific primers and human telomere-specific primers) (Inqaba Biotec, Pretoria, South Africa), 1 µL of nuclease-free water and 1 µL of template DNA (DNA isolated from the study participants). The human 36B4 and human telomere-specific primer sequences are shown in Table 1. A no template control (contains all PCR components but no template DNA) and a control DNA (a pooled sample of template DNA from 10 randomly selected study participants that was used in all PCR runs) reaction were also set up to determine the sensitivity, specificity, precision and accuracy of the telomere length assay.

The PCR conditions were as follows: (i) Annealing temperature for telomere primers was 56 °C, and human 36B4 was 58 °C. The holding stage for both genes was 95 °C for 10 min. The PCR stages for the telomere gene were as follows: (i) denaturing (95 °C for 30 s), (ii) annealing (56 °C for 1 min) and (iii) extension: (a) stage 1 (95 °C for 30 s), (b) stage 2 (56 °C for 1 min) and (c) stage 3 (95 °C for 30 s). PCR stages for human 36B4 were as follows: (i) denaturing (95 °C for 30 s), (ii) annealing (58 °C for 1 min) and (iii) extension (a) stage 1 (95 °C for 30 s), (ii) stage 2 (58 °C for 1 min) and (iii) stage 3 (95 °C for 30 s).

### 2.5. Quality Control

All participants and controls were run in triplicates. The human 36B4 housekeeping reference gene was used for standardisation of the results. A no-template DNA control sample (calibrator sample) was used in each run to determine the overall reaction specificity and to compare outcomes between runs [33]. For each run, melt curves were produced in order to recognise and validate PCR products. Prior to testing samples, the linearity of the human 36B4 assay and human telomere assay was determined by creating a standard curve using quadruplicates of serially diluted DNA (twofold dilutions: 25 ng/µL, 12.5 ng/µL, 6.25 ng/µL, 3.125 ng/µL and 1.56 ng/µL).

### 2.6. Outcomes and Statistical Analysis

Relative telomere lengths (RTLs) were calculated as described by Joglekar et al. and Cawthon [33,34]. The ratio of the human telomere copy number (T) to the human 36B4 single-copy gene number (S) (T/S ratio) was used to express relative telomere lengths in relation to the mean T/S ratio across all samples. Hence, T/S ratio = [2^Ct^(telomeres)/2^Ct^(*36B4*)]^–1^ = 2^–ΔCt^.

All statistical analyses were performed by using Stata Statistical Software: Release 17 (College Station, TX, USA: StataCorp LLC. StataCorp. 2019) and GraphPad Prism 5 (GraphPad Software, Inc., San Diego, CA, USA) statistical software packages. A *p*-value less than 0.05 was considered statistically significant. The Kruskal–Wallis test and Dunn’s multiple-comparison test were used to examine some of the demographic and clinical data; the results were shown as the median (25th–75th percentiles). All socio-demographic parameters (gender, water source, toilet use, previous worm infection and helminth species prevalence were presented as the n (%) and analysed using the Chi-squared test. The data comparing differences in telomere length between groups (in Section 3.3) are presented as the mean and standard deviation, and data were analysed using ANOVA and the Tukey–Kramer post hoc test.

Multivariate regression analysis was used to assess the association between relative telomere length and full blood count and biochemical parameters in individuals who were HIV and helminth singly infected and co-infected. The uninfected control group (no helminths or HIV infections) was used as the reference group. Infections are commonly associated with the disruption of blood biochemical and haematological parameters, which inadvertently influence telomere length [35,36]. Likewise, environmental and socio-demographic factors also impact telomere length [37,38]. Therefore, all these will be considered as confounders in the telomere length analysis. Hence, the RTL data was adjusted for the following:

(i) Infection status and socio-demographic factors (age, gender, BMI, HIV status, HIV viral loads, TB infection status, any illness in the past 30 days, previous worm infection, deworming medication in the past 6 months, allergic reaction in the past 30 days, chronic illnesses, chronic medication taken, supplements taken, antibiotics/probiotics taken in the past 2 weeks and antiretroviral therapy), intake of alcohol, drugs, cigarettes or marijuana, employment and income status, education, water source, toilet type and present diseases.

(ii) Blood biochemical parameters (blood iron, ferritin, transferrin, transferrin saturation, calcium, total protein, phosphate, magnesium, vitamin A, albumin, zinc, C-reactive protein and total IgE).

(iii) Full blood count parameters (red cell count, haemoglobin, haematocrit, mean corpuscular volume, mean corpuscular haemoglobin, mean corpuscular haemoglobin concentration, platelets, red cell distribution width, white cell count, neutrophils, lymphocytes, monocytes, eosinophils and basophils).

## 3. Results

### 3.1. Quality Control

The human 36B4 and human telomere PCR assays displayed good linearity with R^2^ = 0.97 (Supplementary Figure S1) and R^2^ = 0.94 (Supplementary Figure S2), respectively. This is in keeping with a study by Joglekar et al. (2020), which found R^2^ = 0.92 for the human telomere PCR assay and R^2^ = 0.93 for the human 36B4 PCR assay [33]. The current study used the same RTL assay protocol.

### 3.2. Demographics and Clinical Characteristics

Table 2 presents the demographics and clinical characteristics of the participants. The median age and BMI of all 200 participants were 39 years and 26 kg/m^2^, respectively. The majority of the participants were females (63%). Over 18% of the participants used public taps, 31% used pit toilets and 11% used flush toilets that were not connected to the sewage. Furthermore, 31% of the participants had a history of previous worm infection, and the majority were presently infected with *A. lumbricoides* (34.5%), followed by *Schistosoma* spp. (3%), *Enterobius* spp. (2%), *Taenia* spp. (2%), *Strongyloides* spp. (2%) and others. Around 5% were infected with protozoan *Entamoeba* spp. 

### 3.3. Relative Telomere Length in HIV and Helminth Singly Infected and Co-Infected Participants

**Figure 1:**
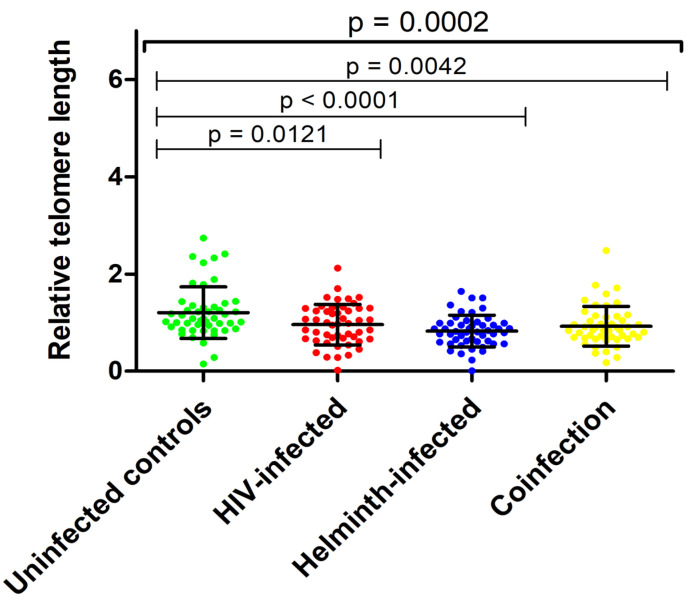
Relative telomere length in HIV and helminth singly infected and co-infected groups.

The uninfected control group had the highest mean RTL (1.21 ± 0.53) while the HIV-infected (0.96 ± 0.42) and co-infected (0.93 ± 0.41) groups had similar RTLs, and lastly, the helminth-infected (0.83 ± 0.33) group had the lowest RTL (*p* = 0.0002). More specifically, the control group had significantly higher RTL compared to the HIV-infected (*p* = 0.0121), helminth-infected (*p* < 0.0001) and co-infected (*p* = 0.0042) groups (Figure 1).

### 3.4. RTL Adjusted for Confounders, Full Blood Counts and Biochemical Parameters

(i) The HIV-infected (β = −0.47, *p* = 0.022), helminth-infected (β = −0.57, *p* = 0.000) and HIV and helminth co-infected (β = −0.45, *p* = 0.047) groups had significantly shorter RTL compared to the uninfected control group (reference group) when the RTL was adjusted for socio-demographic parameters only. (ii) The helminth-infected group (β = −0.37, *p* = 0.008) had a significantly shorter RTL compared to the uninfected control group when the RTL was adjusted for biochemical parameters and socio-demographic parameters. (iii) The helminth-infected (β = −0.62, *p* = 0.000) and HIV and helminth co-infected (β = −0.58, *p* = 0.031) groups had significantly shorter RTLs compared to the uninfected control group when the RTL was adjusted for full blood counts and socio-demographic parameters. (iv) The helminth-infected group (β = −0.51, *p* = 0.004) had significantly shorter RTL compared to the uninfected control group when the RTL was adjusted for biochemical parameters, full blood count and socio-demographic parameters (Table 3).

### 3.5. Multivariate Association of Relative Telomere Length with Biochemical Parameters

When compared to the uninfected control group (reference group), the HIV and helminth co-infected group had significantly shorter RTL when associated with blood iron (β = −0.48, *p* = 0.038), ferritin (β = −0.55, *p* = 0.000), transferrin (β = −0.57, *p* = 0.015), transferrin saturation (β = −0.57, *p* = 0.019), phosphate (β = −0.47, *p* = 0.039), vitamin A (β = −0.49, *p* = 0.050) and C-reactive protein (β = −0.52, *p* = 0.034) levels (Table 4). 

### 3.6. Multivariate Association of Relative Telomere Length with Full Blood Count Parameters

When compared to the uninfected control group (reference group), the HIV and helminth co-infected group had significantly shorter RTL when associated with red cell count (β = −0.44, *p* = 0.052), haemoglobin (β = −0.47, *p* = 0.044), haematocrit (β = −0.46, *p* = 0.045), mean corpuscular volume (β = −0.47, *p* = 0.038), mean corpuscular haemoglobin (β = −0.47, *p* = 0.035), platelets (β = −0.45, *p* = 0.048), red cell distribution width (β = −0.47, *p* = 0.039), lymphocytes (β = −0.45, *p* = 0.048), monocytes (β = −0.45, *p* = 0.048), eosinophils (β = −0.45, *p* = 0.048) and basophils (β = −0.44, *p* = 0.050) (Table 5).

## 4. Discussion

The current study aimed to investigate whether telomere length shortening is accelerated in a South African population co-infected with HIV and helminths compared to those singly infected with either HIV or helminths. HIV and helminth infections were found to be independently and concurrently associated with shortened RTL in this study population. There was a significant association between shorter RTL and biochemical and full blood count parameters in HIV singly infected, helminth singly infected and HIV and helminth co-infected participants. These results demonstrate an association between HIV and helminth co-infection and telomere shortening in the study population, leading to biological ageing [39].

Our study observed shorter RTL in HIV and helminth singly infected and HIV and helminth co-infected participants even after adjusting for confounders. Additionally, females have been reported to have longer telomeres compared to males, but there is limited evidence on the causes of these gender-associated telomere length differences [40]. Previous studies associated HIV infection with shorter telomere length [16,24,41]. A study by [42] reported a significant decline of telomere length in untreated HIV chronic stage and no changes in telomere length during ART.

Currently, there are limited human studies investigating whether helminth infections promote telomere length shortening. Nonetheless, malaria was shown to cause telomere shortening in avian species [43] as well as in humans [27]. Supporting our findings, it has been reported that human helminthiasis, in general, can cause chronic inflammation, genetic instability, and host immunological regulation by disrupting inter- and intracellular communications, disrupting proliferation–anti-proliferation pathways and stimulating the development of malignant stem cells [44]. Helminth infections can also produce oxidative stress, which can result in tissue damage and inflammation [45]. Both processes enhance cell proliferation, which may hasten telomere leukocyte shortening [46,47].

Telomere length shortening can be influenced by several factors, including lifestyle, socioeconomic traits, body mass index (BMI), low physical activities, smoking, psychological stress, infections and diseases [48,49,50]. Additionally, HIV and helminth co-infection has been reported to be associated with anaemia and nutrient deficiency [29]. Given that oxidative stress and inflammation both increase telomere shortening and that nutrition influences both of these processes, it is crucial to further study the association between RTL and blood biochemical parameters in people singly infected and co-infected with HIV and helminths. We observed an association between telomere shortening and C-reactive protein (CRP) in all the infected groups. CRP may be involved in the biochemical process that results from obesity to short telomere length via low-grade inflammation, accelerated cell turnover in the bone marrow and subtle alterations in leukocyte distribution [50].

Blood iron is required for erythrocyte synthesis, enzyme formation and function, immune system functionality and metabolic functions in the human body [51]. Individuals with an excess amount of iron, characterised by higher serum ferritin or transferrin saturation levels, have shorter Leukocyte Telomere Length (LTL) [52]. In middle-aged and older patients, higher serum transferrin saturation concentrations and lower total iron-binding capacity concentrations were associated with shorter LTL [53]. The current study observed a significant association between shorter telomere length (TL) and higher iron levels in the HIV and helminth co-infected group. In addition, a significant association between shorter TL and decreased amounts of transferrin in the HIV and helminth co-infected group was observed.

Magnesium participates in several physiological pathways, including energy production, nucleic acid and protein synthesis, ion transport, cell signalling and structural roles. In the current study, shorter TLs were significantly associated with increased levels of magnesium in all the groups. Calcium can also affect molecular processes, such as gene expression and regulation and protein phosphorylation and modification, cellular functions, such as energy metabolism, and cell division, proliferation, differentiation and death [54]. In our study, shorter TLs were associated with an increase in calcium levels in HIV and helminth singly infected groups but not the HIV and helminth co-infected group. In older females, higher plasma calcium and magnesium levels were also associated with shorter TLs [55]. Similar to our study, a study found statistically significant associations between higher serum phosphate levels and longer mean TL, especially in male participants [56]. Although we found no studies on the association of TL with total protein, vitamin A, zinc, albumin and IgE, we found increased levels of total protein, zinc and total IgE to be associated with shorter TL in the HIV and helminth co-infected group. Furthermore, we found decreased levels of vitamin A to be associated with shorter TL in the HIV and helminth co-infected group.

The immune system is extremely susceptible to telomere shortening since its competence is solely dependent on cell renewal and clonal proliferation of T- and B-cell populations [57]. The association of TL and haematological profile in relation to any disease is poorly understood. The current study found an association between TL shortening and full blood count parameters (red blood cell count, haemoglobin, haematocrit, mean corpuscular volume, mean corpuscular haemoglobin, platelets, red cell distribution width, lymphocytes, monocytes, eosinophils and basophils) in HIV and helminth singly infected and HIV and helminth co-infected participants. Shorter TL was significantly associated with decreased red blood cell and white blood cell counts, increased mean corpuscular haemoglobin and mean cell volume. A study by [58] found an association between RTL and the percentage of basophils but no association between RTL and monocyte count, mean cell haemoglobin or red cell distribution width in healthy patients. Furthermore, shorter telomere length was significantly associated with lower white blood cell count (monocyte count, lymphocyte count, neutrophil count, basophil count and eosinophil count) and neutrophils [59]. Lower red blood cell counts, larger mean red blood cell size, increased red cell distribution width, higher haemoglobin levels and lower platelet count were associated with shorter TL [60]. The difference in results found by [58,60] indicates an inconclusive association between TL and full blood counts. Although more research is required to establish the causal association between telomere length and all the full blood counts, it is possible that confounders such as stress and cortisol affecting full blood counts may affect the TL. Additionally, given that blood cell features are implicated in oxidative and inflammatory processes, the association between telomeres and blood cells may be bidirectional [61].

This is the first study to analyse telomere length in helminth singly infected and HIV and helminth co-infected adult participants in South Africa. Furthermore, the study analysed the association between telomere length and biochemical and full blood counts of HIV singly infected, helminth singly infected and HIV and helminth co-infected participants.

The study is limited by a cross-sectional design; therefore, we cannot infer a causal link between these infections and accelerated telomere shortening. Therefore, an association rather than a cause is reported. Secondly, participants were purposefully selected based on HIV and helminth infection status, thus with an inherent selection bias. Furthermore, the association between telomere length shortening and the severity of helminth infections in the study participants was not assessed.

Furthermore, telomerase is an important enzyme for protecting telomere length attrition. Future studies should characterise this enzyme in HIV and helminth singly infected and co-infected individuals in order to determine its potential therapeutic role.

## 5. Conclusions

The current study demonstrated that HIV, helminth and HIV and helminth co-infection are associated with telomere shortening. The phenomenon is known to result in biological ageing. Our results also indicate that both the infections singly and concurrently influence the association between telomere length and HIV and helminth co-infection. The findings add new knowledge on the deleterious effects of dual infection with helminths and HIV in adults. This adds more reasons for advocacy for expanding deworming to include adults in helminth- and HIV-endemic regions.

## Figures and Tables

**Table 1:** Human 36B4 and human telomere-specific primer sequences for QPCR.

Gene	Forward Primer Sequence and Concentration Used	Reverse Primer Sequence and Concentration Used	Reference
Human 36B4 gene	5′-CAGCAAGTGGGAAGGTGTAATCC-3′ (30 pmol/μL)	5′-CCCATTCTATCATCAACGGGTACAA-3′ (30 pmol/μL)	[33]
Human telomere-specific gene	5′-GGGTTTGTTTGGGTTTGGGTTTGGGTTTGGGTTTGGGTTTGGGTT-3′ (30 pmol/μL)	5′-GGCTTGCCTTACCCTTACCCTTACCCTTACCCTT-3′ (30 pmol/μL)	[33]

**Table 2:** Demographic and clinical characteristics of the study participants.

Parameter	All Participants (n = 200)	Uninfected Controls (n = 50)	HIV-Infected (n = 50)	Helminth-Infected (n = 50)	HIV + Helminth Co-Infection (n = 50)	*p*-Value
**Age (years)**	39 (30–50)	43 (26.8–58.3)	41.00 (33.8–49)	34.5 (27–53.8)	38.5 (30.8–45)	0.33
**Gender, n (%):**						0.1245
**Male**	74 (37)	22 (44)	18 (36)	22 (44)	12 (24)	
**Female**	126 (63)	28 (56)	32 (64)	28 (56)	38 (76)	
**BMI (kg/m^2^)**	26 (22–33)	28.2 (23.4–34.5)	25.65 (20.38–31.6)	25.4 (23.6–32.7)	24.9 (22.6–31.1)	0.056
**Water source, n (%)**						0.1725
Own tap—inside and outside	153 (76.5)	42 (84)	41 (82)	34 (68)	36 (72)	
Public tap	36 (18)	5 (10)	8 (16)	14 (28)	9 (18)	
Others	11 (5.5)	3 (6)	1 (2)	2 (4)	5 (10)	
**Toilet use, n (%)**						0.0888
Pit toilets	62 (31)	10 (20)	16 (32)	13 (26)	23 (46)	
Flush toilets connected to sewage	116 (58)	36 (72)	27 (54)	31 (62)	22 (44)	
Flush toilets not connected to sewage	22 (11)	4 (8)	7 (14)	6 (12)	4 (8)	
**Previous worm infection**						
Yes	62 (31)	11 (22)	14 (28)	18 (36)	19 (38)	
No	138 (69)	39 (78)	36 (72)	32 (64)	31 (62)	
**CD4+ count (cells/µL)**	730 (505–970)	896 (708–1169)	541 (267.8–728.5)	861 (712.3–1066)	654.5 (388.5–810.3)	0.2800
**Viral load (copies/mL)**			20 (20–180)		20 (20–132)	0.6376
<20 copies/mL, n (%)			35 (70)		34 (68)	1.000
>20 copies/mL, n (%)			15 (30)		16 (32)	
**Helminth species, n (%)**						
*Ascaris lumbricoides*	69 (34.5)			32 (64)	37 (74)	
*Schistosoma* spp.	6 (3)			3 (6)	3 (6)	
*Enterobus* spp.	4 (2)			1 (2)	3 (6)	
*Hymenolepis* spp.	1 (0.5)			1 (2)	0 (0)	0.0001
*Taenia* spp.	4 (2)			3 (6)	1 (2)	
*Trichuris* spp.	1 (0.5)			1 (2)	0 (0)	
*Strongyloides* spp.	4 (2)			2 (4)	2 (4)	
Hookworm spp.	1 (0.5)			1 (2)	0 (0)	
**Protozoa species, n (%)**						
*Entamoeba* spp.	10 (5)			6 (12)	4 (8)	

**Table 3:** Multivariate association of relative telomere length with HIV and helminth-infected and uninfected control groups.

	Unstandardised β–Coefficient Values (Reference Group: Uninfected Controls)					
Parameters	HIV-Infected		Helminth-Infected		HIV and Helminth Co-Infected	
	β	*p*-Value	β	*p*-Value	Β	*p*-Value
Unadjusted RTL	−0.35 (−0.59–−0.11)	0.004	−0.49 (−0.72–−0.26)	0.000	−0.39 (−0.63–−0.15)	0.002
Adjusted RTL 1 (Adjusted for socio-demographic parameters) ^(i)^	−0.47 (−0.86–−0.07)	0.022	−0.57 (−0.84–−0.30)	0.000	−0.45 (−0.89–0.01)	0.047
Adjusted RTL 2 (Adjusted for socio-demographic and biochemical parameters) ^(ii)^	−0.34 (−0.82–0.13)	0.153	−0.37 (−0.64–−0.10)	0.008	−0.09 (−0.66–0.48)	0.737
Adjust RTL 3 (Adjusted for socio-demographic and full blood count parameters) ^(iii)^	−0.46 (−0.92–0.01)	0.055	−0.62 (−0.90–−0.33)	0.000	−0.58 (−1.11–−0.05)	0.031
Adjust RTL 4 (Adjusted for socio-demographic, biochemical and full blood count parameters) ^(iv)^	−0.32 (−0.93–0.28)	0.276	−0.51 (−0.84–−0.18)	0.004	−0.35 (−1.05–0.35)	0.308

Note: ^(i)^ Adjusted RTL 1: Adjusted for socio-demographic parameters (age, gender, BMI, HIV status, employment, income source/occupation monthly income, education, intake of alcohol per day, intake of drugs or marijuana, water source, toilet type, present diseases aware of, any illness in the past 30 days, previous worm infection, deworming medication in the past 6 months, allergic reaction in the past 30 days, chronic illness, medication taken, supplements taken, antibiotics/probiotics taken in the past 2 weeks, TB infected and antiretroviral therapy). ^(ii)^ Adjusted RTL 2: Adjusted for socio-demographic parameters above for adjusted RTL 1 and biochemical parameters (blood iron, ferritin, transferrin, transferrin saturation, calcium, total protein, phosphate, magnesium, vitamin A, albumin, zinc, C-reactive protein and total IgE). ^(iii)^ Adjusted RTL 3: Adjusted for confounders mentioned above for adjusted RTL 1 and full blood count parameters (red cell count, haemoglobin, haematocrit, mean corpuscular volume, mean corpuscular haemoglobin, mean corpuscular haemoglobin concentration, platelets, red cell distribution width, white cell count, neutrophils, lymphocytes, monocytes, eosinophils and basophils). ^(iv)^ Adjusted RTL 4: Adjusted for socio-demographic parameters mentioned above for adjusted RTL 1, full blood count parameters and biochemical parameters.

**Table 4:** Multivariate association of relative telomere length with biochemical parameters in HIV and helminth infected and uninfected control groups.

	Unstandardised β–Coefficient Values (Reference Group: Uninfected Controls)						
Parameters		RTL in HIV-Infected		RTL in Helminth-Infected		RTL in HIV and Helminth Co-Infected	
		β (95% CI)	*p*	β (95% CI)	*p*	β (95% CI)	*p*
Iron (µmol/L)	A	−0.36 (−0.60–−0.12)	0.003	−0.49 (−0.72–−0.26)	0.000	−0.40 (−0.64–−0.16)	0.001
	B	−0.47 (−0.87–−0.07)	0.022	−0.58 (−0.85–−0.31)	0.000	−0.48 (−0.93–−0.03)	0.038
Ferritin (ng/mL)	A	−0.35 (−0.59–−0.11)	0.005	−0.46 (−0.70–−0.22)	0.000	−0.41 (−0.66–−0.16)	0.002
	B	−0.48 (−0.88–−0.07	0.022	−0.55 (−0.84–−0.25)	0.000	−0.48 (−0.95–−0.00)	0.048
Transferrin (g/L)	A	−0.35 (−0.59–0.11)	0.005	−0.45 (−0.69–−0.22)	0.000	−0.40 (−0.64–−0.16)	0.002
	B	−0.48 (−0.89–−0.08)	0.019	−0.54 (−0.83–−0.25)	0.000	−0.57 (−1.03–−0.11)	0.015
Transferrin saturation (%)	A	−0.36 (−0.60–−0.11)	0.004	−0.47 (−0.71–−0.23)	0.000	−0.41 (−0.66–0.16)	0.001
	B	−0.47 (−0.87–−0.07)	0.023	−0.57 (−0.86–−0.27)	0.000	−0.57 (−1.05–−0.10)	0.019
Calcium (mmol/L)	A	−0.35 (−0.59–−0.10)	0.005	−0.49 (−0.72–−0.25)	0.000	−0.38 (−0.62–−0.13)	0.003
	B	−0.47 (−0.87–−0.08)	0.020	−0.57 (−0.84–−0.31)	0.000	−0.40 (−0.85–0.05)	0.078
Total protein (g/L)	A	−0.37 (−0.62–−0.11)	0.005	−0.46 (−0.69–−0.22)	0.000	−0.38 (−0.63–−0.14)	0.002
	B	−0.48 (−0.90–−0.06)	0.026	−0.54 (−0.84–−0.25)	0.000	−0.44 (−0.92–0.33)	0.068
Phosphate (mmol/L)	A	−0.36 (−0.60–−0.12)	0.004	−0.49 (−0.72–−0.26)	0.000	−0.39 (−0.63–−0.15)	0.002
	B	−0.51 (−0.91–−0.11)	0.014	−0.57 (−0.84–−0.30)	0.000	−0.47 (−0.92–−0.03)	0.039
Magnesium (mmol/L)	A	−0.40 (−0.64–−0.15)	0.002	−0.53 (−0.76–−0.30)	0.000	−0.39 (−0.63–−0.14)	0.002
	B	−0.52 (−0.91–−0.13)	0.010	−0.64 (−0.92–−0.36)	0.000	−0.39 (−0.85–0.06)	0.088
Vitamin A (µg/L)	A	−0.33 (−0.58–−0.08)	0.011	−0.48 (−0.72–−0.24)	0.000	−0.38 (0.64–−0.11)	0.006
	B	−0.4 (−0.81–0.02)	0.060	−0.58 (−0.86–−0.29)	0.000	−0.49 (−0.98–−0.00)	0.050
Albumin (g/L)	A	−0.09 (−0.29–0.11)	0.375	−0.27 (−0.44–−0.09)	0.004	−0.14 (−0.33–0.06)	0.177
	B	−0.29 (−0.65–0.07)	0.110	−0.30 (−0.53–−0.06)	0.016	−0.11 (−0.56–0.33)	0.611
Zinc (µmol/L)	A	−0.32 (−0.57–−0.07)	0.012	−0.48 (−0.71–−0.25)	0.000	−0.37 (−0.63–−0.12)	0.005
	B	−0.49 (−0.89–−0.08)	0.021	−0.58 (−0.85–−0.31)	0.000	−0.43 (−0.92–0.06)	0.086
C-Reactive Protein (mg/L)	A	−0.35 (−0.59–−0.11)	0.005	−0.47 (−0.70–−0.23)	0.000	−0.39 (−0.64–−0.15)	0.002
	B	−0.47 (−0.87–−0.07)	0.022	−0.54 (−0.84–−0.25)	0.001	−0.52 (−1.00–−0.04)	0.034
Total IgE (KU/L)	A	−0.34 (−0.58–−0.10)	0.006	−0.46 (−0.70–−0.23)	0.000	−0.37 (−0.61–−0.13)	0.003
	B	−0.45 (−0.86–−0.05)	0.030	−0.57 (−0.85–−0.29)	0.000	−0.44 (−0.89–0.02)	0.059

Note: (i) A: Unadjusted data (dependent variable is RTL, and independent variable is the biochemical parameter of interest). (ii) B: Adjusted data (dependent variable is RTL, and independent variable is the biochemical parameter of interest and socio-demographic parameters).

**Table 5:** Multivariate association of relative telomere length with full blood count parameters in HIV and helminth infected and uninfected control groups.

	Unstandardised β–Coefficient Values (Reference Group: Uninfected Controls)						
Parameters		RTL in HIV-Infected		RTL in Helminth-Infected		RTL in HIV and Helminth Co-Infected	
		β (95% CI)	*p*	β (95% CI)	*p*	β (95% CI)	*p*
Red cell count (10^12^/L)	A	−0.36 (−0.62–−0.10)	0.006	−0.49 (−0.72–−0.26)	0.000	−0.39 (−0.64–−0.15)	0.002
	B	−0.45 (−0.87–−0.04)	0.033	−0.56 (−0.83–−0.29)	0.000	−0.44 (−0.89–0.00)	0.052
Haemoglobin (g/dL)	A	−0.36 (−0.61–−0.11)	0.004	−0.48 (−0.71–−0.25)	0.000	−0.39 (−0.62–−0.15)	0.002
	B	−0.45 (−0.86–−0.05)	0.029	−0.57 (−0.84–−0.31)	0.000	−0.47 (−0.93–−0.01)	0.044
Haematocrit (L/L)	A	−0.37 (−0.62–−0.13)	0.004	−0.48 (−0.72–−0.25)	0.000	−0.38 (−0.62–−0.14)	0.002
	B	−0.47 (−0.87–−0.06)	0.025	−0.57 (−0.84–−0.30)	0.000	−0.46 (−0.92–−0.01)	0.045
Mean corpuscular volume (fL)	A	−0.35 (−0.59–−0.11)	0.005	−0.50 (−0.73–−0.27)	0.000	−0.45 (−0.70–−0.19)	0.001
	B	−0.46 (−0.86–−0.06)	0.025	−0.57 (−0.84–−0.30)	0.000	−0.47 (−0.91–−0.03)	0.038
Mean corpuscular haemoglobin (pg)	A	−0.35 (−0.59–−0.11)	0.005	−0.50 (−0.73–−0.27)	0.000	−0.45 (−0.71–−0.20)	0.001
	B	−0.47 (−0.87–−0.07)	0.022	−0.58 (−0.85–−0.31)	0.000	−0.47 (−0.91–−0.03)	0.035
Mean corpuscular haemoglobin concentration (g/dL)	A	0.35 (−0.59–−0.11)	0.005	−0.49 (−0.72–−0.26)	0.000	−0.39 (−0.64–−0.15)	0.002
	B	−0.47 (−0.87–−0.07)	0.022	−0.59 (−0.86–−0.32)	0.000	−0.44 (−0.89–−0.01)	0.055
Platelets (10^9^/L)	A	−0.35 (−0.59–−0.11)	0.005	−0.48 (−0.71–−0.25)	0.000	−0.37 (−0.62–−0.13)	0.003
	B	−0.46 (−0.86–−0.06)	0.025	−0.57 (−0.84–−0.30)	0.000	−0.45 (−0.89–−0.00)	0.048
Red cell distribution width (%)	A	−0.36 (−0.60–−0.11)	0.005	−0.49 (−0.72–−0.26)	0.000	−0.42 (−0.66–−0.18)	0.001
	B	−0.46 (−0.86–−0.06)	0.026	−0.56 (−0.83–−0.29)	0.000	−0.47 (−0.93–−0.02)	0.039
White cell count (10^9^/L)	A	−0.36 (−0.60–0.11)	0.005	−0.49 (−0.72–−0.25)	0.000	−0.38 (−0.64–−0.12)	0.005
	B	−0.48 (−0.88–−0.08)	0.020	−0.56 (−0.84–−0.29)	0.000	−0.44 (−0.90–−0.02)	0.059
Neutrophils (10^9^/L)	A	−0.35 (−0.59–−0.11)	0.005	−0.48 (−0.72–−0.25)	0.000	−0.38 (−0.63–−0.12)	0.004
	B	−0.48 (−0.88–−0.08)	0.020	−0.55 (−0.83–−0.28)	0.000	−0.44 (−0.90–−0.03)	0.064
Lymphocytes (10^9^/L)	A	−0.36 (−0.61–−0.11)	0.004	−0.49 (−0.72–−0.26)	0.000	−0.39 (−0.63–−0.14)	0.003
	B	−0.47 (−0.86–−0.07)	0.023	−0.58 (−0.85–−0.31)	0.000	−0.45 (−0.90–−0.01)	0.048
Monocytes (10^9^/L)	A	−0.36 (−0.60–−0.11)	0.004	−0.48 (−0.71–−0.25)	0.000	−0.40 (−0.64–−0.15)	0.002
	B	−0.47 (−0.88–−0.08)	0.020	−0.57 (−0.84–−0.30)	0.000	−0.45 (−0.89–−0.00)	0.048
Eosinophils (10^9^/L)	A	−0.35 (−0.60–−0.11)	0.004	−0.49 (−0.72–−0.26)	0.000	−0.38 (−0.63–−0.14)	0.002
	B	−0.47 (−0.88–−0.07)	0.022	−0.57 (−0.84–−0.30)	0.000	−0.45 (−0.90–−0.00)	0.048
Basophils (10^9^/L)	A	−0.36 (−0.60–−0.12)	0.004	−0.49 (−0.72–−0.26)	0.000	−0.38 (−0.62–−0.14)	0.002
	B	−0.51 (−0.91–−0.11)	0.014	−0.57 (−0.83–−0.30)	0.000	−0.44 (−0.88–−0.00)	0.050

Note: (i) A: Unadjusted data (dependent variable is RTL, and independent variable is the full blood count parameter of interest). (ii) B: Adjusted data (dependent variable is RTL, and independent variable is the full blood count parameter of interest and socio-demographic parameters).

## Data Availability

Data generated from this study can be accessed from the corresponding author upon reasonable request.

## Supplementary Materials

The following supporting information can be downloaded at: https://www.mdpi.com/article/10.3390/cimb46070409/s1, Supplementary Figure S1: Human 36B4 gene standard curve. Supplementary Figure S2: Human telomere gene standard curve.
