# Supplementary material for: Telomere Length in a South African Population Co-Infected with HIV and Helminths
Source: Curr Issues Mol Biol. 2024 Jul 2;46(7):6853–67. doi: 10.3390/cimb46070409 (PMC11276263; doi:10.3390/cimb46070409)
Supplement: Supplementary file 1 [file cimb-46-00409-s001.zip › cimb-3048725-supplementary.pdf]

SUPPLEMENTARY Figures: Standard Curve for quality control

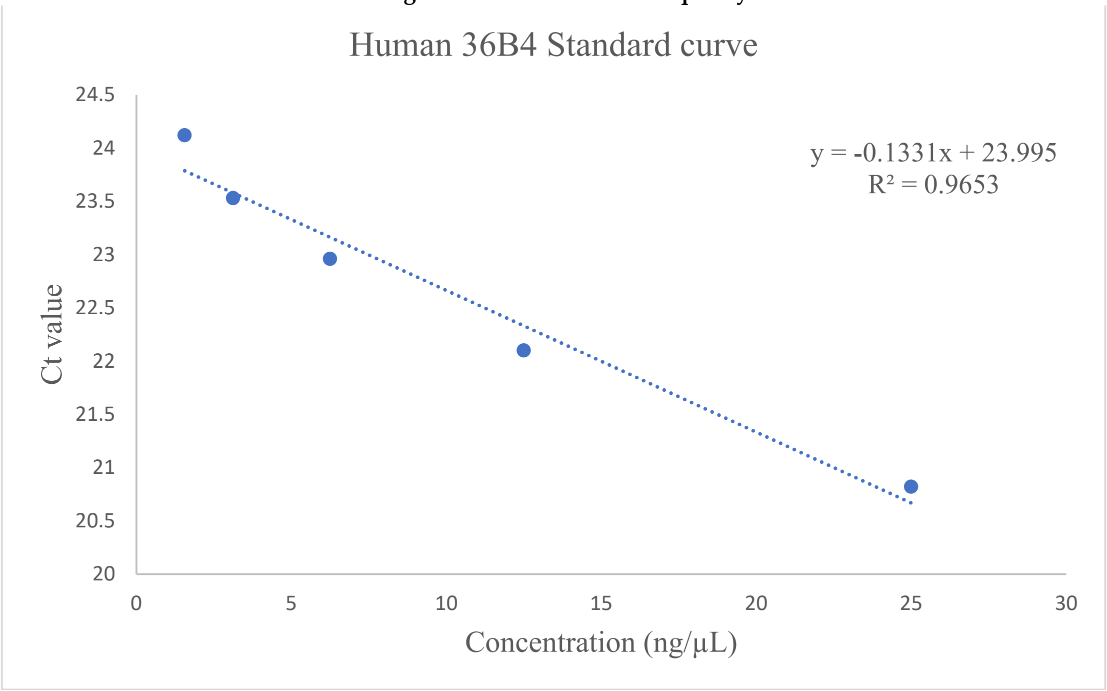

Supplementary Figure S1: Human 36B4 gene standard curve.

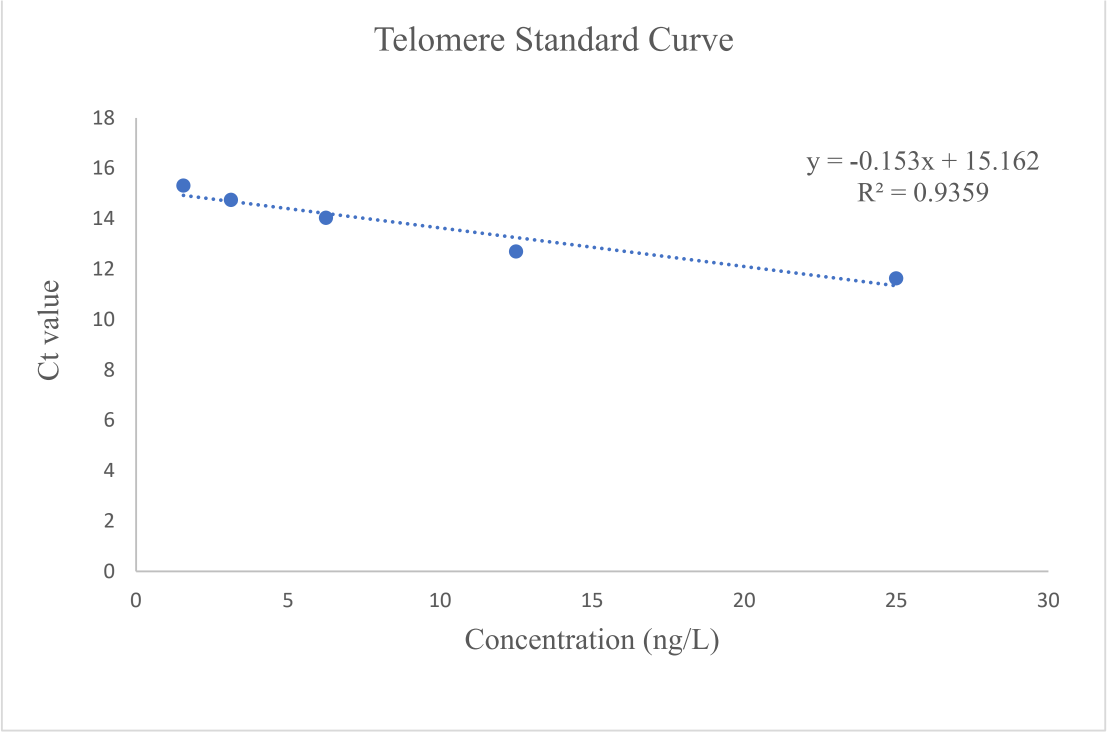

Supplementary Figure S2: Human telomere gene standard curve.
